# A Rare Case of Intra-Articluar Osteoid Osteoma of the Elbow Managed With Arthroscopic Excision

**DOI:** 10.7759/cureus.15666

**Published:** 2021-06-15

**Authors:** Yassin M Alrassasi, Meshal A Almustafa, Zainab M Al Eid, Mustafa Y Albattat, Khaleel I Al Batran

**Affiliations:** 1 Orthopaedic Surgery and Sports Medicine, King Fahad Hospital Hofuf, Alhasa, SAU; 2 Orthopaedic Surgery, King Fahad Hospital Hofuf, Alhasa, SAU

**Keywords:** olecranon, osteoid, osteoma, arthroscopy, elbow

## Abstract

Osteoid osteoma (OO) of the elbow is a very rare entity as it typically occurrs in the lower extremity of extra-articular long bones. Hereby, we report a case for a 28-year-old female patient has been diagnosed with a unique presentation of sub-periosteal osteoid osteoma in the intra-articular portion of distal humerus. She underwent arthroscopic excision for the lesion which is considered a reliable minimally invasive management modality. In addition to this, findings of a 1-year follow-up after excision is reported.

## Introduction

Osteoid osteoma (OO) was first described by Jaffe in 1935 as a benign osteoblastic tumor. These lesions are composed of two elements, the periosteal reaction and the nidus that contained the osteoid osteoma itself [1-2]. The nidus commonly manifests as an oval with minor diameter not exceeding 1 cm [1]. Osteoid osteomas once present, are usually found in the diaphysis of the extra-articular long bones such as the femur and the tibia [3]. Intra-articular osteoid osteoma within or near a joint is very rarely present, and is hence considered as a distinct entity [2-4]. Furthermore, occurrence in the distal humerus and elbow joint is uncommon and is observed in only up to 5-12% of all diagnosed osteoid osteomas [4-7]. Once these lesions present in the elbow, it manifests with atypical clinical features and radiological findings that are completely different from lesions presented in the extra-articular portions [2].

Classically, patients complaining of nocturnal pain usually respond to non-steroidal anti-inflammatory drugs, especially salicylates [4-6]. In contrast, intra-articular lesions show minimal reactive cortical thickening or sclerosis which results in difficulty to determine imaging findings accurately, which causes a delay in the diagnosis [8]. The precise diagnostic approach of intra-articular lesions remains challenging. Thus, these lesions easily mimic other differentials such as monoarticular arthritis and tendonitis [9-12]. Once the diagnosis is made, management is a must as these lesions can be excised through various recognized modalities such an open approach, computed tomography- (CT-) guided excision and radio-frequency ablation [10-11]. Recently, excision through arthroscopy has gained popularity as a minimally-invasive option, especially for lesions proximal to the neurovascular structures [7-8]. A limited number of intra-articular osteoid osteoma cases at elbow joint have been published in literature [8-9]. There are arthroscopic maneuvers possible to do debridement and release of the capsule which can aid in enhanced the range of motion [13]. In addition to this, limited reports about arthroscopic removal of osteoid osteoma of elbow have been described [7-9, 14]. In our paper, we present a unique case of arthroscopic excision of osteoid osteoma in the distal humerus that was treated successfully with marvelous outcomes.

## Case presentation

**Figure 1:**
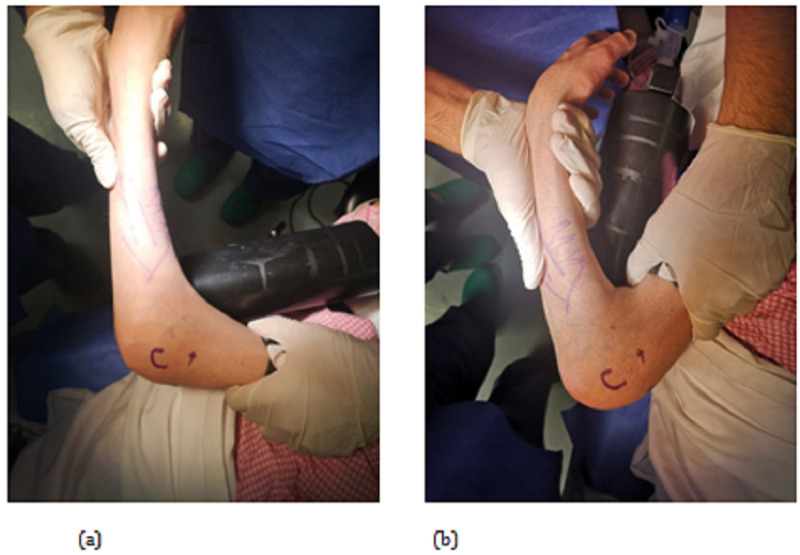
Preoperative assessment ranges of motion (ROM). Elbow extension (a) and flexion (b).

A 28-year old right-hand dominant female patient presented to our outpatient orthopedic clinic with a history of elbow pain that exacerbates at night and improved with NSAIDs. A limited range of motion (ROM) of the elbow joint was the main concern for her visit. Physical examination had shown a contracture and ROM between 50-110° which is demonstrated in figure 1.

**Figure 2:**
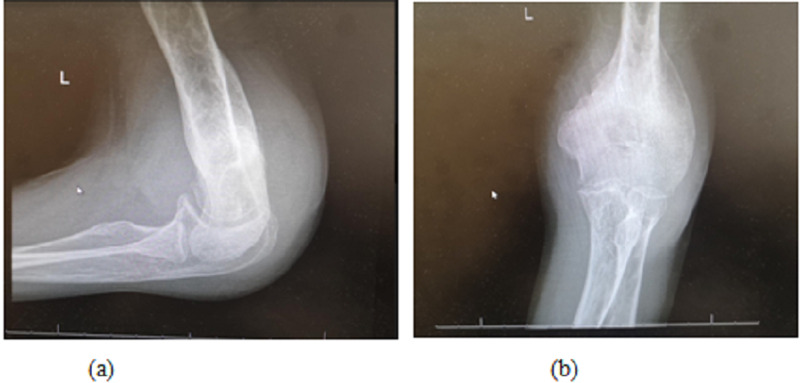
Preoperative Radiographical Images of Elbow lateral view (a) and anteroposterior view (b)

Radiographical images show enormous periosteal reaction at the distal of humerus as shown in figure 2.

**Figure 3:**
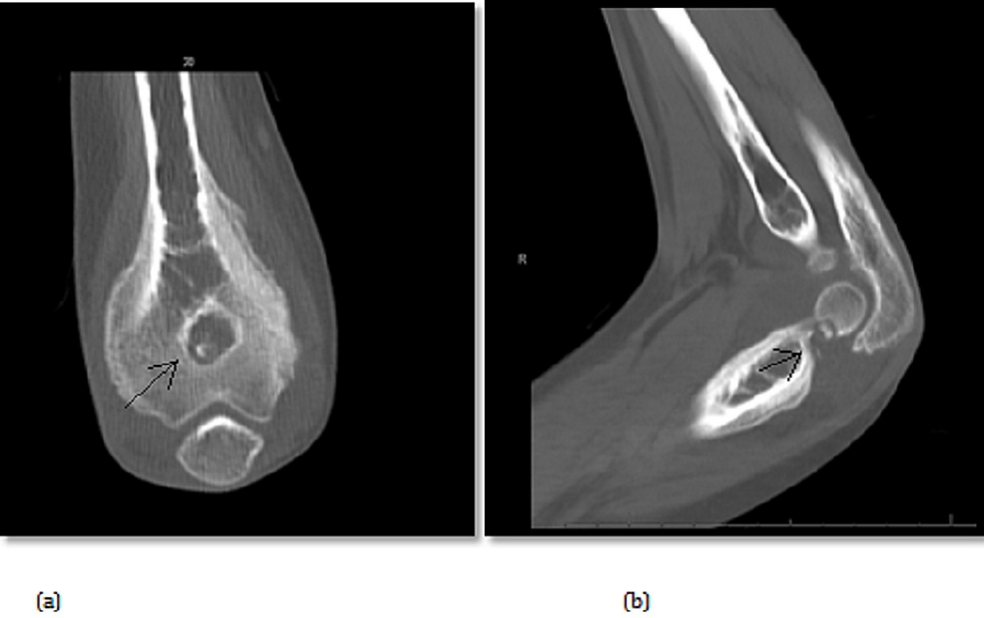
Preoperative computed tomography images [coronal (a) and sagittal (b), respectively] demonstrates a nidus comprising central sclerosis.

This was followed by conducting a CT scan which showed typical findings of osteoid osteoma and nidus located in the olecranon fossa associated with significant periosteal reaction and joint effusion (Figure 3).

**Figure 4:**
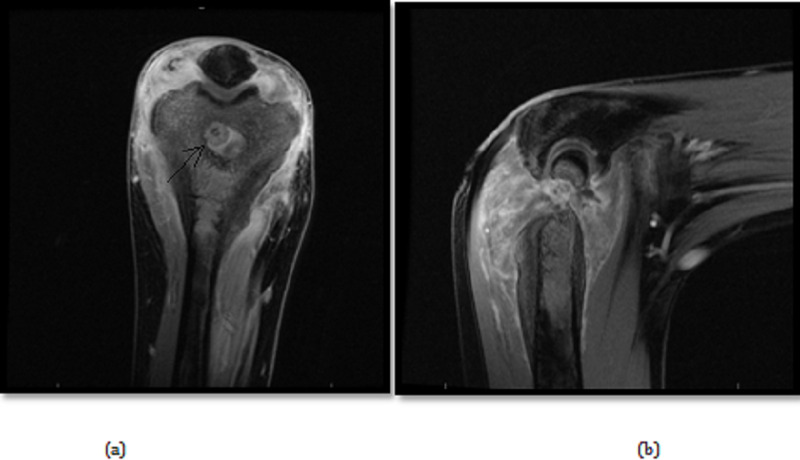
Preoperative magnetic resonance imaging (coronal (a) and sagittal (b), respectively)

Furthermore, MRI showed a cortical based intra-articular distal humeral osteoid osteoma complicating the joint with severe effusion, synovitis and soft tissue reactive changes with bone marrow edema as seen in figure 4. Thus, features are in keeping with cortical based intra-articular distal humeral osteoid osteoma

The patient was taken for surgery under general anesthesia. The surgeon choose arthroscopy as the modality for excision and removal of nidus associated with synovectomy. Three portals was made: anteromedial, anterolateral and posterior.

**Figure 5:**
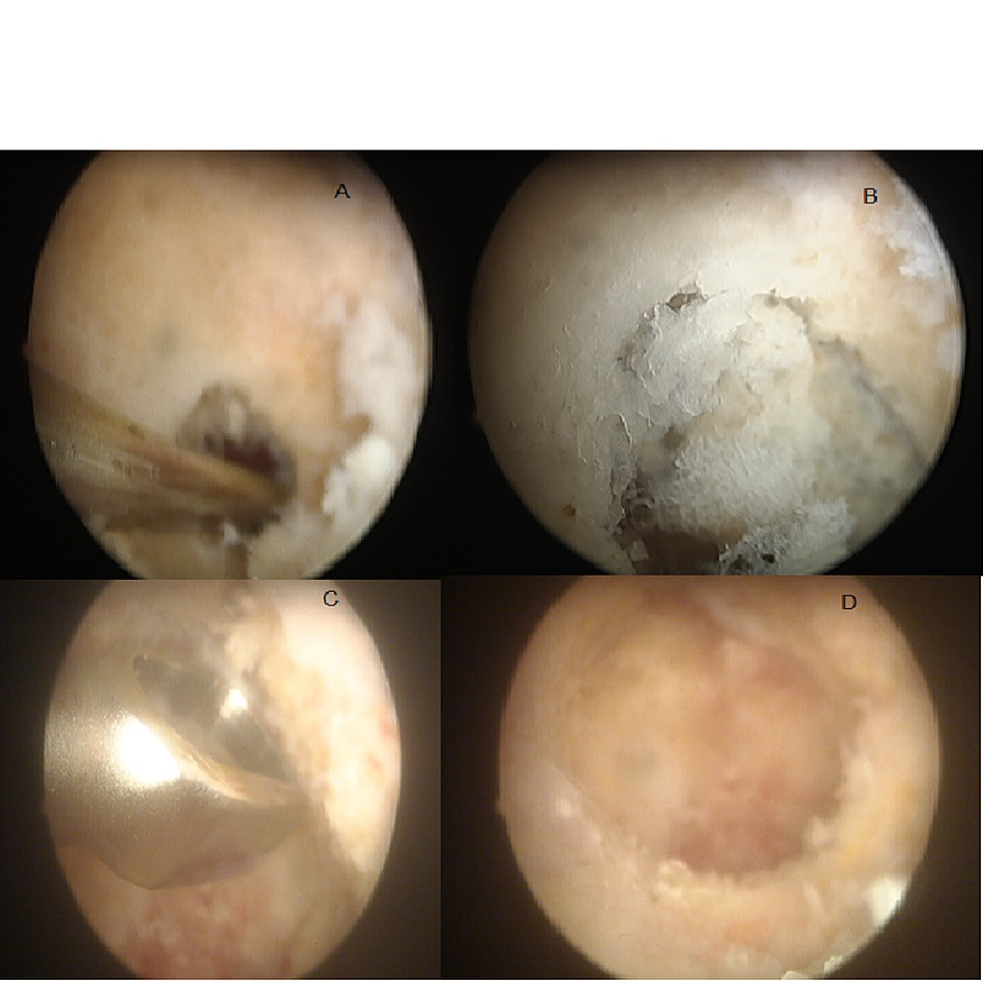
: Arthroscopic findings , show surface of osteoid osteoma was hyperemic (a,b,c,d)

Enormous proliferation of synovial tissue was observed and shaving was made through the posterior portal. Olecranon fossa was identified and the surface of osteoid osteoma was hyperemic as shown in figure 5. The nidus was excised through the posterior portal and sent to histopathology; the margin then was debrided with the shaver and ablation device. The range of motion post-op was examined and it showed almost full flexion.

**Figure 6:**
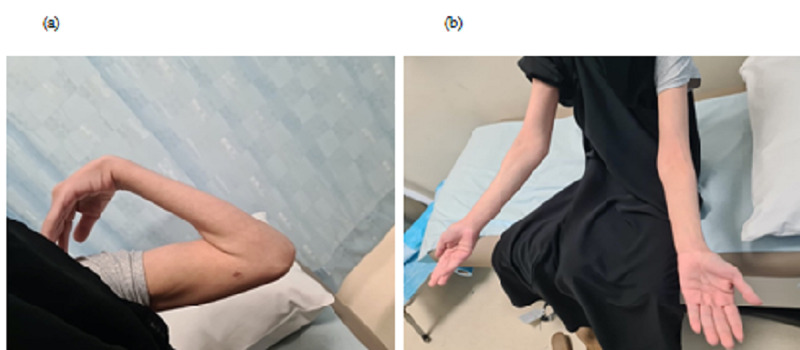
Ranges of motion at one-year follow-up. Elbow flexion (a) and extension (b)

**Figure 7:**
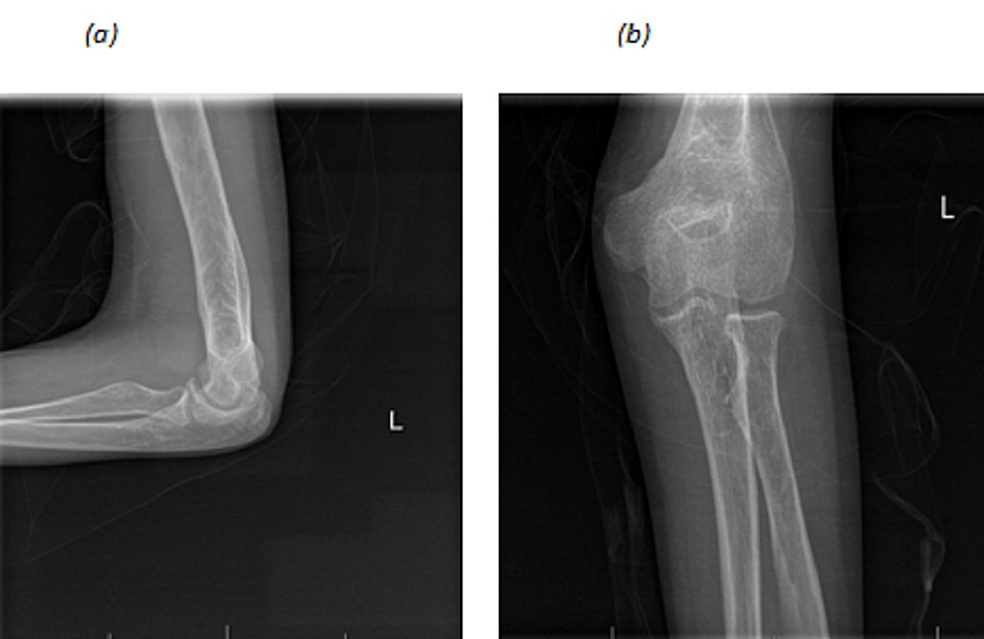
Periosteal reaction almost subside after 1 year lateral view (a) anteroposterior view (b)

Upon follow up after 1 year of the surgery, there was no recurrence of the lesion, and symptoms (i.e. nocturnal pain) subsided completely. Also, range of motion improved to 30-145°. The patient is completely satisfied about the result (Figure 6), and the periosteal reaction subsided almost completely (figure 7).

## Discussion

Osteoid osteoma is known to be one of the benign bone tumours. The findings of plain radiograph imaging typically show presence of a radiolucent nidus associated with reactive bone sclerosis which commonly identified in the diaphysis of long bones and extremely rare in intra-articular site [15-18]. In the literature, authors report various locations where osteoid osteoma has been identified, including lesions in the proximal ulna, coronoid process of the ulna, in the capitellum and distal humerus [16,18]. Thus, misdiagnosis of these lesions as tenosynovitis, synovitis or arthritis is attributed to its rare identification in the intra-articular sites. Furthermore, such diagnosis of osteoid osteoma in the joints such as in the elbow can be delayed and overlooked due to inflammatory and infectious monarthritis, or stress fracture and other various differential diagnosis which might be more common. However, osteoid osteoma typically responds to NSAIDs and aspirin with exacerbating of pain at night, which assists in diagnosis. This would be attributed to presence of E2 prostaglandin which is suppressed by anti-inflammatory agents. In the intra-articular location, the response of NSAIDs for pain relief is less effective in comparison to the extra-articular sites [17]. Such classical presentation of worsening pain at night and relief by aspirin or non-steroidal anti-inflammatory drugs facilitate the recognition of osteoid osteoma.

In the literature is a case series which included 17 patients and 5 case reports more for patients for whom surgeons performed minimal invasive arthroscopic treatment for elbow OOs [2, 9-10,14-17]. Furthermore, outcomes were fairly reasonable with satisfactory outcomes; however, residual symptoms attributed to improper removal of the lesions among a patient that osteoid osteoma presented in the anterior aspect of trochlea [16].

 There are unique advantages of performing the excision arthroscopically like decreased risk of infection, better cosmetic outcomes and less post-operative pain, leading to faster healing and return to work with reasonable range of motion.

In the literature, few cases have reported the arthroscopic excision of juxta-articular and intra-articular osteoid osteomas in the elbow [18-19]. Modality of arthroscopy resulted in proper visualization of joint space that facilitates identification of nidus.

Kamrani et al. published the results of treating osteoid osteoma of the elbow through arthroscopic ablation in 10 patients which concluded that diagnosis was still not confirmed after arthroscopic excision due to insufficiency of sample that was taken [17]. In addition, Zupanc et al. reported arthroscopic excision of a 42-year-old male patient who presented with symptomatic lesion at the capitellum. The excision of the nidus was performed arthroscopically with a good view of joint space [9].

When publishing the outcomes of their patient, the authors noticed that flexion contracture that existed after removing the nidus is resolved spontaneously with no further intervention needed. However, patients develop remarkable restriction in the range of motion [15-16].

## Conclusions

Herein, we report a case for intra-articular osteoid osteoma of the elbow whilst demonstrating the successful outcomes of using minimally invasive arthroscopic approach for excision of such a tumor in the elbow joint.
